# Mitochondrial tRNA^Ser(UCN)^ mutations associated non-syndromic sensorineural hearing loss in Chinese families

**DOI:** 10.1016/j.heliyon.2024.e27041

**Published:** 2024-03-06

**Authors:** Dejun Zhang, Jie Wu, Yongyi Yuan, Xiaohong Li, Xue Gao, Dongyang Kang, Xin Zhang, Sha-sha Huang, Pu Dai

**Affiliations:** a Department of Otolaryngology Head and Neck Surgery, The Second Hospital of Jilin University, Changchun, China; b ColIege of Otolaryngology Head and Neck Surgery, Chinese PLA General Hospital, Beijing, China; c State Key Lab of Hearing Science, Ministry of Education, National Clinical Research Center for Otolaryngologic Diseases, Beijing, China; d Beijing Key Lab of Hearing Impairment for Prevention and Treatment, Beijing, China; e Department of Otolaryngology, Head and Neck Surgery, National Children's Medical Center/Beijing Children's Hospital, Capital Medical University, Beijing, China; f Department of Otolaryngology, PLA Rocket Force Characteristic Medical Center, Beijing, China

**Keywords:** Hearing loss, Mitochondria, Transfer RNA, Non-syndromic, Transfer RNA^Ser(UCN)^

## Abstract

Mitochondrial transfer RNA mutation is one of the most important causes of hereditary hearing loss in humans. Mitochondrial transfer RNA^Ser (UCN)^ gene is another hot spot for mutations associated with non-syndromic hearing loss, besides the 12S ribosomal RNA gene. In this study, we assessed the clinical phenotype and the molecular characteristics of two Chinese families with non-syndromic hearing loss. Mutational analysis revealed that 7445A > G and 7510T > C mutations in the mitochondrial transfer RNA^Ser (UCN)^ gene were the molecular etiology of Family 1 and Family 2, respectively. However, the clinical and genetic characteristics of the two families carrying the above mutations in the transfer RNA^Ser (UCN)^ gene exhibited a variable expression of hearing loss and an incomplete penetrance. Sequencing analysis of the complete mitochondrial genome showed the presence of transfer RNA^Trp^ 5568A > G and NADH-ubiquinone oxidoreductase chain 4 11696G > A mutations in Family 1. The mitochondrial haplotype analysis showed that the two families belonged to Asian D4 and M80′D haplotypes, respectively, and no pathogenic variations were found in the nuclear genes. To our knowledge, our study is the first to report 7445A > G and 7510T > C mutations in the mitochondrial transfer RNA^Ser (UCN)^ gene, in multi-generation non-syndromic hearing loss pedigrees from China. Our study suggests that 5568A > G and 11696G > A mutations may enhance the penetrance of hearing loss in Chinese Family 1, while mitochondrial haplotypes and known nuclear genes may not be modifiers for the phenotypic expression of 7445A > G and 7510T > C mutations in these Chinese families.

## Highlights

• We report 7445A > G and 7510T > C mutations in the mitochondrial tRNA^Ser (UCN)^ gene in non-syndromic hearing loss pedigrees.
• m.5568A > G and m.11696G > A mutations may enhance the penetrance of hearing loss in the Chinese Family harboring m.7445A > G.
• Mitochondrial haplotypes and known nuclear genes may not be modifiers for the phenotypic expression in our Chinese families.
• Aminoglycoside may play a potential role in increasing the susceptibility of individuals with m.7510T > C mutation to SNHL.

## 1 Introduction

Mitochondria are organelles found in most eukaryotic cells, and are responsible for energy production for an organism's physiological activities [1]. They have their own genetic material, the mitochondrial DNA (mtDNA). Mitochondrial genetic disorders can affect one or more tissues or organs, especially those organs which require high energy such as the brain, heart and cochlea. Sensorineural hearing loss (SNHL) caused by mutations in the mtDNA, is a typical mitochondrial genetic disorder, which plays an important role in hereditary hearing loss [2].

The human transfer RNA^Ser (UCN)^ (tRNA^Ser (UCN)^) gene is located at the cytosine-rich light (L) strand of mtDNA and encodes serine^(UCN)^-carrying tRNA. Mutations in the tRNA^Ser (UCN)^ gene changes the secondary structure of tRNA, which affects the synthesis of mitochondrial protein and the production of ATP, and finally leads to mitochondrial dysfunction and deafness [3]. The prevalence of tRNA^Ser (UCN)^ gene associated non-syndromic SNHL was found to be low in the Chinese population. In the two studies carried out by Jin et al. and Tang et al. reporting on the large scale (1452 subjects, 2651 subjects, respectively) mutational screening of tRNA^Ser (UCN)^ gene, they did not find any 7445A > G and 7510T > C mutations [4,5]. In the current study, we performed mutational analysis in two Chinese families with non-syndromic SNHL. Two pathogenic mitochondrial tRNA^Ser(UCN)^ mutations: 7445A > G and 7510T > C were identified in the two families respectively. However, the clinical and genetic evaluation showed a high variability in the penetrance and expression of the hearing loss phenotype in the two families with the tRNA^Ser (UCN)^ mutations, and in other similar reported families. To further investigate the potential role of modifier genes in the manifestation of the variable phenotypes being observed, we sequenced and analyzed the complete mitochondrial genome and the related nuclear genes.

## 2 Materials and methods

### 2.1 Families and clinical evaluation

Two Chinese families with non-syndromic SNHL (Family 1 and Family 2) were recruited in our study. Comprehensive clinical information including the subjective degree of hearing loss, age of onset, medication, noise exposure, pathological changes in the ear and other relevant clinical manifestations, were obtained using a questionnaire. The degree of hearing loss was classified into: mild (25–40 dB HL); moderate (41–55 dB HL); moderately severe (56–70 dB HL); severe (71–90 dB HL); and profound (>90 dB HL) hearing loss, based on the WHO (1997) criteria.

The ethics committee of the Chinese People's Liberation Army (PLA) General Hospital (approval number S2016-120-02) approved the study design, and written informed consent to publish was obtained from the participants or the parents of the minor subjects.

### 2.2 Deafness gene panel sequencing

Genomic DNA was isolated from the peripheral blood samples of the participating members from the two families using a gDNA blood extraction kit (Qiagen, Valencia, CA, USA) within 1 week of sample collection. A deafness gene panel including 159 nuclear deafness genes, and 6 mitochondrial genes (Supplementary Table 1) was applied to the proband and part of the related family members from the two families（V:12 in Family 1, II:11, II:12 and IV:1 in Family 2). We performed capture sequencing and next-generation sequencing (NGS) of the coding exons of the deafness genes and their flanking 100 bps on an Illumina HiSeq 2000 from MyGenostics Corporation (Beijing China). The pipeline was followed as described previously by Yuan et al. [6]. In brief, the gDNA was fragmented, ligated to adaptors, extracted, amplified by ligation-mediated PCR and then all coding exons and the 100 bp flanking of the 168 genes were captured using the GenCap Custom Exome Enrichment kit (MyGenostics, Beijing, China). Each captured library was loaded onto the Illumina Hiseq 2000 platform. After filtering out low-quality and duplicate reads, clean data were aligned to the human reference genome hg19 using the Burrows-Wheeler Aligner (BWA). After alignment, all variants were annotated and classified using the Genome Analysis Toolbox (GATK), Variant Effect Predictor (VEP). Afterwards, variants were further annotated using the following public databases.Exome Aggregation Consortium (ExAC); the 1000 Genomes Project; dbSNP (v.144); Genome Aggregation Database (gnomAD); ClinVar; the Human Gene Mutation Database (HGMD), and Deafness Variation Database (http://deafnessvariationdatabase.org/). Pathogenicity of variants were predicted using the PolyPhen-2, SIFT, MutationTaster and CADD programs. The variants interpreted by the ACMG/AMP guidelines for genetic hearing loss.

### 2.3 Mutational analysis of the complete mtDNA and related nuclear genes

The genomic DNA of the matrilineal relatives from the two families (II:3, IV:10, IV:19 and V:12 in Family 1; II:1, II:3, II:5, II:7, II:11, II:13, III:5, III:8, III:12 and IV:1 in Family 2) were amplified by polymerase chain reaction (PCR). NGS of the whole mtDNA genome and the related nuclear genes was performed, and then DNA sequence analysis was performed for the patients as described above (**2.2)**. To identify mutations within the obtained genome, the sequences were compared with the revised Cambridge reference sequence (GenBank accession number: NC_012920) and haplogroup phylogeny from MITOMAP (http://www. mitomap.org).

### 2.4 Sanger sequencing

Genomic DNA was extracted from peripheral blood of maternal members of two families. Sanger sequencing was performed to validate the filtered candidate variants and examine co-segregation of the genotype and phenotype. The primers for PCR amplification of m.7445A > G and m.7510T > C were: Forward, 5′-TTTCTTCCCACAACACTT-3′ and reverse, 5′-AGGAAATAGAAACCGTCTGA-3′.

## 3 Results

### 3.1 Clinical characteristics of the two families with SNHL

Our study investigated two SNHL pedigrees in Chinese families. The segregation pattern of the hearing loss phenotype was consistent with matrilineal transmission of hearing loss (Fig. 1A and Fig. 2A). In Family 1, the proband (V:12) was a 9-year-old boy who began to suffer bilateral hearing loss at birth. Since then, the hearing loss had gradually increased and was accompanied by tinnitus. His physical development was normal and he had no intellectual disability. In addition to the clinical manifestations of bilateral SNHL, the uncle of the proband also suffered from palmoplantar keratoderma. In the other matrilineal family members, the degree of hearing loss ranged from normal hearing to profound SNHL, with or without tinnitus, and the time of onset ranged from birth to adulthood (Fig. 1B and Supplementary Table 2). Among all the 32 matrilineal members, 29 suffered from SNHL and 5 matrilineal members also suffered from palmoplantar keratoderma (Fig. 1C). The penetrance of SNHL was 91% (29/32). None of the family members belonging to Family 1 had a history of using Aminoglycoside antibiotics (AmAn).

**Fig. 1A:**
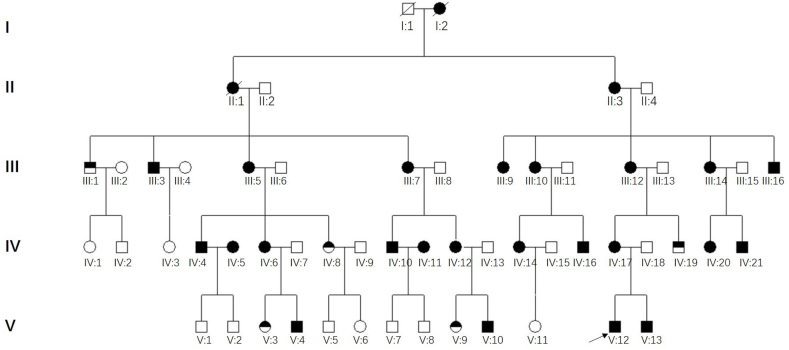
Pedigree of Family 1. The Roman symbols on the left indicate the generation. The filled in symbols indicate individuals affected with SNHL. The half-filled in symbols indicate individuals affected by both SNHL and palmoplantar keratoderma. Males are represented by squares and females by circles. The blank symbols denote normal or near normal hearing or those with status unknown. An arrow denotes the proband.

**Fig. 1B:**
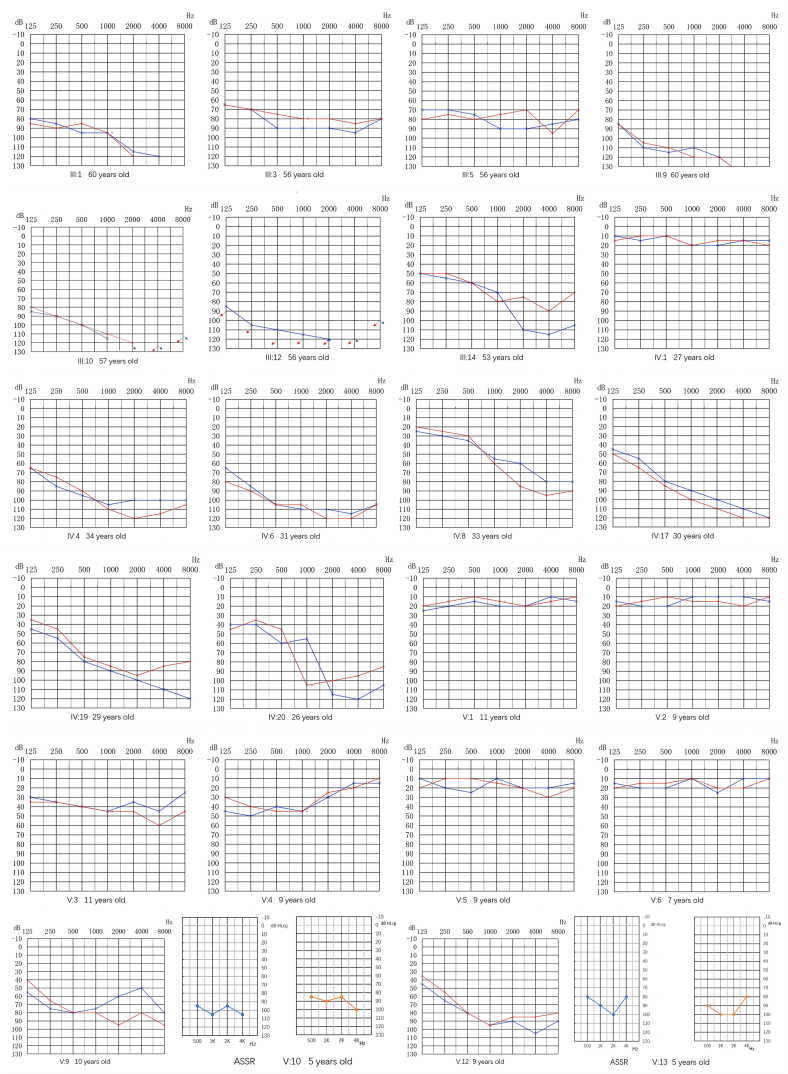
Audiograms of Family 1. Hearing levels of the right and the left ear are marked using red and blue lines, respectively. The blue or red arrows indicate no response to audiometric stimulation. The Roman numeral below the audiograms represents their identity in the pedigree and the numeral in parenthesis the age of the subjects.

**Fig. 1C:**
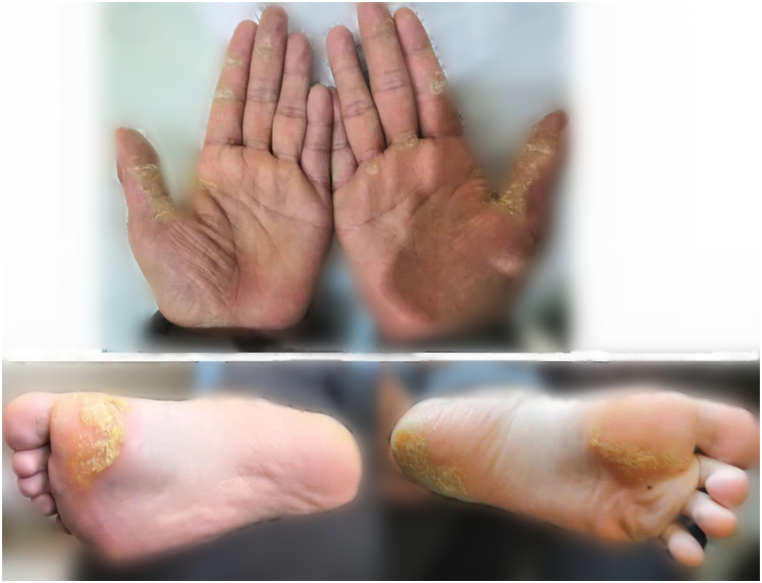
Palmar and plantar keratoderma. Some members of the Family1 present with bilateral keratinization of the palms and plantars. (For interpretation of the references to colour in this figure legend, the reader is referred to the Web version of this article.)

In the Family 2 (Fig. 2A), the proband (IV:1) was a 24-year-old female with hearing loss. At the age of 6 years, she suffered bilateral hearing loss and tinnitus after receiving a streptomycin injection. Upon visiting a doctor at the local hospital, she was diagnosed with bilateral severe SNHL. There was no significant improvement in her hearing ability after treatment with vasoactive drugs and corticosteroids. Part of the subjects (II:5, II:12, II:13, III:5, III:11 and IV:1) underwent detailed physical examination and examination by the otorhinolaryngology specialist. No other positive signs were found except that pure tone audiometry showed SNHL (Fig. 2B and Supplementary Table 3). The penetrance rate of hearing loss in Family 2 was 61% (11/18).

**Fig. 2A:**
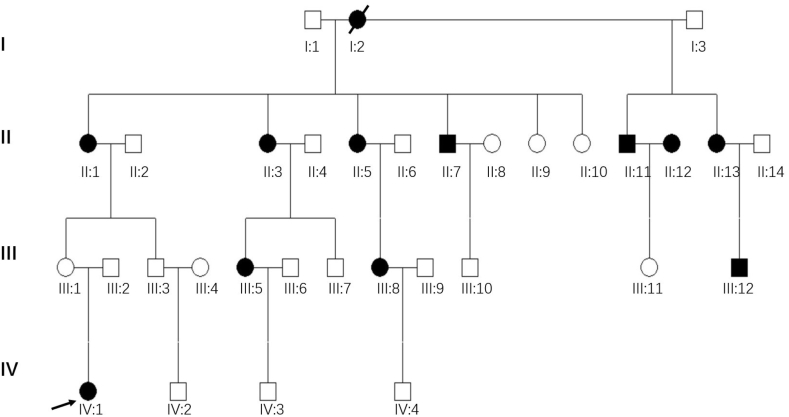
Pedigree of Family 2. The Roman symbols on the left indicate the generation. The filled in symbols indicate individuals affected with SNHL. Males are represented by squares and females by circles. The blank symbols denote normal or near normal hearing or those with status unknown. An arrow denotes the proband.

**Fig. 2B:**
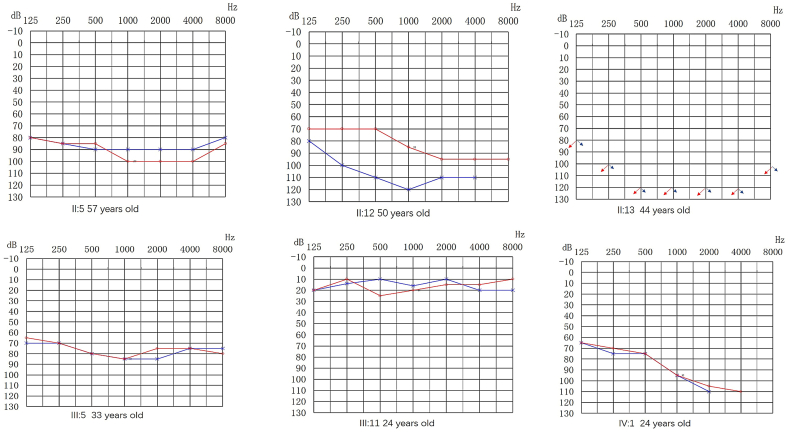
Audiograms of some members in Family 2. Hearing levels of the right and the left ear are marked using red and blue lines, respectively. The blue or red arrows indicate no response to audiometric stimulation. The Roman numeral below the audiograms represents their identity in the pedigree and the numeral in parenthesis the age of the subjects. (For interpretation of the references to colour in this figure legend, the reader is referred to the Web version of this article.)

### 3.2 Deafness gene panel sequence analysis

Using the deafness gene panel, we sequenced all the coding exons and about 100 bp of the flanking intronic sequence of selected patients from the two families. A homoplasmic m.7445A > G mutation in the Family 1 and a homoplasmic m.7510T > C mutation in the Family 2 were identified and further confirmed in their matrilineal family members by Sanger sequencing. Additionally, a compound heterozygous mutation, GJB2 c.299_300delAT/c.35dupG, was identified in II:13 of Family 2.

### 3.3 Mutational analysis of the complete mtDNA and the related nuclear genes

To investigate the role of mitochondrial polymorphisms and the potential role of other related nuclear genes in the clinical phenotype observed in the two families, we amplified and sequenced the complete mtDNA and related nuclear genes of the selected patients, and compared the sequencing results with the revised Cambridge Reference Sequences. In addition to the CO1/tRNA^Ser (UCN)^ 7445A > G (haploproup D4) and tRNA^Ser (UCN)^ 7510T > C (haploproup M80′D) mutations, we also found tRNA^Thr^ 5568A > G and ND4 11696G > A mutations in Family 1. A number of polymorphic sites were identified in the two families. Among them, 3 were newly identified mutation sites, including 5760G > C, 6857C > G and 15428G > C (Table 1). No positive mutations were found in the nuclear gene sequencing results.

**Table 1:** MtDNA mutations in the two Chinese families with hearing loss.

Position	Gene locus	Nucleotide change	rCRS	Amino acid change	Conservation (H/B/M/X)	Family1	Family 2	Allele frequency^a^	Pathogenicity^a^	Reported^b^
7445	COI/tRNASer^(UCN)^	A > G	A		A/A/A/A	G		0/3.54e-5	Pathogenic	YES
7510	tRNA^Ser(UCN)^	T > C	T		T/T/T/T		C	0/0	Pathogenic	YES
73	D-Loop	A > G	A			G	G	7.16e-1/1.07e-4		YES
150	D-Loop	C > T	C				T	1.66e-1/6.57e-4		YES
200	D-Loop	A > G	A				G	3.60e-2/7.10e-4		YES
263	D-Loop	A > G	A			G	G	9.91e-1/1.95e-4		YES
302	D-Loop	A > ACCCC	A				ACCCC			YES
310	D-Loop	T > C	T			C				YES
398	D-Loop	T > A	T				A	4.08e-4/0		YES
489	D-Loop	T > C	T			C	C	1.09e-1/3.55e-5		YES
545	D-Loop	G > C	G				C	1.24e-4/7.09e-5		YES
750	12S rRNA	A > G	A		A/A/G/-	G	G	9.83e-1/1.06e-4		YES
752	12S rRNA	C > T	C		C/C/A/-		T	6.91e-4/0		YES
1107	12S rRNA	T > C	T		T/C/T/T		C	1.49e-3/1.77e-5	Benign	YES
1410	12S rRNA	G > A	G		G/T/A/T		A			YES
1438	12S rRNA	A > G	A		A/A/A/G	G	G	9.56e-1/7.12e-5	Benign	YES
2706	16S rRNA	A > G	A		A/G/A/A	G	G	7.39e-1/8.93e-5		YES
3010	16S rRNA	G > A	G		G/A/A/A	A		1.63e-1/7.10e-5		YES
3702	ND1	A > G	A		A/A/A/A		G	2.48e-4/0		YES
4769	ND2	A > G	A	Met 100 Met	M/M/M/I	G	G	9.84e-1/3.55e-5	Benign	YES
4883	ND2	C > T	C	Pro 138 Pro	P/P/P/P	T	T	1.09e-2/1.77e-5		YES
5178	ND2	C > A	C	Leu237Met	L/T/T/T	A	A	1.07e-2/0	Benign	YES
5301	ND2	A > G	A	Ile 278 Val	I/L/M/L		G	2.43e-3/0	Benign	YES
5568	tRNA Trp	A > G	A		A/A/G/T	G		2.30e-4/1.77e-5	Benign	YES
5738	OL	G > C	G				C			YES
5760	OL	G > C	G				C			NO
6857	CO1	C > G	C	Val318Val	V/V/V/V		G	8.86e-5/0		NO
7028	CO1	C > T	C	Ala 375 Ala	A/A/A/A	T	T	7.49e-1/3.60e-5	Benign	YES
8414	ATPase8	C > T	C	Leu 17 Phe	L/F/M/W	T		8.98e-3/0	Benign	YES
8701	ATPase6	A > G	A	Thr 59 Ala	T/S/L/Y	G	G	3.03e-1/1.24e-4	Benign	YES
8860	ATPase6	A > G	A	Thr 112 Ala	T/A/A/T	G	G	9.94e-1/1.60e-4	Benign	YES
9180	ATPase6	A > G	A	Val 218 Val	V/V/V/L		G	3.15e-3/8.86e-5		YES
9540	COIII	T > C	T	Leu 112 Leu	L/L/L/L	C	C	3.04e-1/1.78e-5		YES
9575	COIII	G > C	G	Pro 123 Pro	P/P/P/P		C	3.08e-3/1.77e-5		YES
10397	ND3	A > G	A	Trp 113 Trp	W/W/W/W		G	2.13e-3/3.54e-5		NO
10398	ND3	A > G	A	Thr 114 Ala	T/T/S/A	G	G	4.18e-1/1.42e-4	Benign	YES
10400	ND3	C > T	C	Thr 114 Ala	T/T/S/A	T	T	5.28e-2/1.77e-5		YES
10873	ND4	T > C	T	Pro 38 Pro	P/S/S/S	C	C	3.05e-1/0		YES
11218	ND4	C > T	C		T/T/T/T	T		1.77e-5/0		YES
11696	ND4	G > A	G	Val 313 Ile	V/T/T/M	A		9.92e-4/0	Benign	YES
11719	ND4	G > A	G	Gly320Gly	G/G/G/G	A	A	7.11e-1/5.35e-		YES
11944	ND4	T > C	T	Leu395Leu	L/L/L/L		C	8.41e-2/3.55e-5		YES
12026	ND4	A > G	A	Ile 423 Val	I/I/M/L		G	1.08e-3/1.77e-5	Benign	YES
12630	ND5	G > A	G	Trp 98 Trp	W/W/W/W	A		8.12e-3/2.66e-4		YES
12705	ND5	C > T	C	Ile 123 Ile	I/I/I/T	T	T	4.89e-1/4.37e-5		YES
13759	ND5	G > A	G	Ala 475 Thr	A/T/T/I		A	1.49e-2/1.77e-4	Benign	YES
14668	ND6	C > T	C	Met 2 Met	M/M/N/I	T		9.75e-3/5.32e-5		YES
14766	Cytb	C > T	C	Ile 7Thr	T/S/T/S	T	T	7.07e-1/5.35e-5	Benign	YES
14783	Cytb	T > C	T	Leu 13 Leu	L/I/I/I	C	C	5.53e-2/5.33e-5	Likely pathogenic	YES
15043	Cytb	G > A	G	Gly 99 Gly	G/G/G/G	A	A	7.88e-2/1.60e-4	Likely pathogenic	YES
15301	Cytb	G > A	G	Leu 185 Leu	L/L/L/L	A	A	2.45e-1/5.35e-5	Likely pathogenic	YES
15326	Cytb	A > G	A	Thr 194 Ala	T/M/I/I	G	G	9.93e-1/2.13e-4	Benign	YES
15394	Cytb	T > C	T	Asp 216 Asp	D/D/D/D		C	5.85e-4/3.54e-5		YES
15428	Cytb	G > C	G		D/D/D/D		C			NO
16179	D-Loop	CA > C	CA				C	1.51e-3/2.34e-2		YES
16183	D-Loop	A > C	A				C	4.70e-3/1.01e-1		YES
16189	D-Loop	T > C	T				C	2.49e-1/3.93e-3		YES
16209	D-Loop	T > C	T				C	2.90e-2/1.95e-4		YES
16223	D-Loop	C > T	C			T	T	3.94e-1/5.15e-4		YES
16311	D-Loop	T > C	T			C		2.19e-1/2.51e-3		YES
16360	D-Loop	C > T	C				T	3.07e-2/2.48e-4		YES
16362	D-Loop	T > C	T			C	C	1.50e-1/9.78e-4		YES
16398	D-Loop	G > A	G			A		2.85e-3/1.77e-5	Pathogenic	YES

a The Genome Aggregation Database(gnomAD)(http://www.gnomad-sg.org).

b Human MITOMAP database (www.mitomap.org); OL, L-strand origin; Cytb Cytochrome *b*; rCRS, revised Cambridge Reference Sequences; H, human; B, bovine; M, mouse; X, Xenopus laevis; ND, NADH-ubiquinone oxidoreductase chain; CO, cytochrome *c* oxidase; CytB, Cytochrome B; ATP6, ATP synthase membrane subunit 6; Ser, serine; Trp, Tryptophan; Met, Methionine; Pro, Proline; Leu, Leucine; IIe, Isoleucine; Val, Valine; Ala, Alanine; Gly, Glycine; Thr, Threonine; Homo/hetero, Homoplastic/heteroplastic

## 4 Discussion

Mitochondrial tRNA plays an important role in cellular protein synthesis. It is highly conserved in the process of genetic evolution, and abnormal tRNA usually leads to the emergence of genetic diseases. Mutations in the gene encoding tRNA^Ser (UCN)^, leading to a reduction in the tRNA level, have been closely associated with non-syndromic SNHL. In the present study, we investigated the molecular and genetic features of two Chinese families suffering from hearing loss. Using NGS and Sanger sequencing, we identified two pathogenic mutations: 7445 A > G and 7510T > C, located in the tRNA^Ser (UCN)^ gene.

The 7445A > G mutation identified in Family 1 is a well-known abnormality in the precursor of tRNA^Ser (UCN)^ gene, which is associated with hearing loss. The mutation changes the stop codon (AGA) into AGG in the H-strand mRNA, encoded by the mitochondrial COI [3]. At the same time, this mutation is adjacent to the 3’ end site of the endonucleolytic processing of the L-strand RNA precursor [7]. Thus, the 7445A > G mutation significantly reduces the steady-state levels of tRNA^Ser (UCN)^ and decreases the rate of ND6 synthesis, leading to mitochondrial dysfunction, which is a prerequisite for the onset of hearing loss [3]. In terms of the symptoms, the patients harboring 7445A > G mutation not only presented with isolated SNHL, but some patients also presented in combination with palmoplantar keratoderma. Based on the published studies (Table 2), along with our results, we found that palmoplantar keratosis occurred in New Zealand, Japan, France, Portugal and Chinese families (5/10) [[8], [9], [10], [11], [12], [13], [14], [15]]. It can be seen that m.7445A > G mutation could not just damage the cochlear function, but also often induce skin keratosis dysfunction.

**Table 2:** Clinical features of different families with the 7445A > G mutation.

Family	Age at onset(year)	Status of mutation	Penetrance	PPK	Additional mutations	Haplogroup	Country	Reference
**1**	3–18	heteroplasmic	33% (13/39)	–	polymorphism	–	UK	8
**2**	childhood	heteropalsmic	77% (24/31)	PPK	T4216C, T10084C ， G13708A	–	New Zealand	2
**3**	childhood	homoplasmic	60%	PPK	–	–	Japan	9
**4**	>5	homoplasmic	44% (4/9)	PPK	not test	–	France	10
**5**	6–15	homoplasmic	100% (5/5)	_	GJB2 c.35 delG heterozygous	–	UK	11
**6**	–	homoplasmic	100% (11/11)	PPK	not test	–	Portugal	12
**7**	childhood	heteroplasmic	15% (2/13)	–	polymorphisms	U4b	Hungary	13
**8**	>2	homoplasmic	58% (7/12)	–	polymorphisms	H6	Poland	14
**9**	0–10	heteropalasmic	100% (5/5)	–	GJB2, c.235delC heterozygous	–	Japan	15
**10**	0–20	homoplasmic	91% (29/32)	PPK	A5568G,G11696A	D4	China	Our study

PPK, palmoplantar keratoderma.

The second mutation in the mitochondrial genome, the homoplasmic 7510T > C mutation, was previously reported in several families worldwide, from different ethnic backgrounds [[16], [17], [18], [19]]. Structurally, the 7510T > C mutation is located at the 5′ side of the acceptor stem of tRNA and disrupts the highly conserved secondary structure of tRNA^Ser (UCN)^, which may reduce the levels of mature tRNA^Ser (UCN)^ and decrease the synthesis of mitochondrial proteins, causing mitochondrial dysfunction [19]. In the published reports, symptoms associated with the 7510T > C mutation mainly presented as isolated SNHL [[16], [17], [18], [19]], whereas patients from one Finnish family have also presented with neurological symptoms in addition to SNHL [20].

The clinical phenotypes of mitochondrial tRNA^Ser (UCN)^ mutation associated non-syndromic SNHL exhibit variable penetrance and expression within and among families from different ethnic backgrounds. For example, the penetrance of the hearing loss phenotype caused by m.7445 A > G mutation ranged from 15% to 100% across different families, while the penetrance of the hearing loss phenotype caused by m.7510 T > C mutation was 61%–100% (Tables 2 and 3). Therefore, some authors proposed that the modifier factors, such as nuclear modifier genes, mtDNA polymorphisms, mtDNA haplotypes or environmental factors, may contribute to the variable penetrance and expression of SNHL [21]. To investigate whether these modifier factors played a role in the Chinese families in our study, we further performed sequencing analysis of the complete mtDNA and related nuclear genes. The results showed that all maternal members in the Family 1 carried the tRNA^Trp^ 5568A > G and ND4 11696G > A mutations. The 5568A > G mutation was previously found in a family with a multi-generation hearing loss in the UK and was thought to be associated with non-syndromic SNHL [22]. And the 11696G > A mutation could have enhanced the penetrance of deafness-associated 12S rRNA 1555A > G mutation in the Chinese families [23]. Therefore, we speculated that 5568A > G and 11696 G > A mutations may be related to the high degree of penetrance of hearing loss in Family 1, but further studies are needed to confirm this hypothesis. In the Family 2, we found three new polymorphic loci and several known polymorphisms, which were unlikely to affect the phenotypic presentation of SNHL in the family.

**Table 3:** Clinical features of different families with the 7510T > C mutation.

Family	Age at onset(year)	Status of mutaion	Penetrance	Additional mutations	Haplogroup	Country	Reference
1	1.5–70	heteropalasmic	100% (11/11)	polymorphism	–	UK	16
**2**	<20	homoplasmic	84% (24/26)	polymorphism	H1	Spanish	17
**3**	2–40	homoplasmic	100%（16/16）	polymorphism	H	North America	18
**4**	5–20	homoplasmic	83% (5/6)	polymorphism	H	Hungary	19
**5**	After birth-40	homoplasmic	61%（11/18）	polymorphism	M80′D	China	Our study

Furthermore, we did not find mutations in the common nuclear modifier genes, such as *TRMU, MTO1, GTPBP3* and *YARS2* [24], in both the families. Analysis of the mtDNA haplotype demonstrated that the two families belonged to the Eastern Asian haplogroup D4 and M80′D respectively, which was different from the haplogroups of the previously reported families. It indicated that the 7445A > G and 7510T > C mutations occurred through recurrent origins and founder events. The above results suggested that the nuclear modifier genes and mitochondrial genetic background may not play a significant role in the expression of the hearing loss phenotype in the Chinese families in our study [25]. However, the proband in Family 2 had a history of aminoglycoside exposure, which suggests that aminoglycosides may increase the susceptibility of individuals with m.7510T > C mutation to SNHL.

In the present study, we describe two families with matrilineal transmission of non-syndromic SNHL, attributable to the 7445A > G and 7510T > C mutations in the mitochondrial tRNA^Ser (UCN)^ gene. To our knowledge, this is the first study reporting the above two mutations in multi-generation non-syndromic SNHL pedigrees from China. Our study suggests that the tRNA^Trp^ 5568A > G and ND4 11696G > A mutations may play a role in the clinical development of hearing loss in the first family harboring 7445A > G mutation. Furthermore, the nuclear modifier genes and mitochondrial genetic background may not play a significant role in the expression of the hearing loss phenotype in the two families in our study. Finally, aminoglycoside may play a potential role in increasing the susceptibility of individuals with m.7510T > C mutation to SNHL. However, some limitations have to be considered in our study. The use of ordinal NGS for mitochondrial genome sequence has limitations in detecting large-scale mtDNA deletions. We have not yet performed relevant cellular function experiments to validate our conjecture.

## Data availability statement

The data that support the findings of this study are openly available in SRA (Sequence Read Archive) database, accession number [PRJNA1022316].

## Funding

This work was supported by the National Key 10.13039/100006190Research and Development Project, [grant numbers. 2016YFC1000700, 2016YFC1000704, 2016YFC1000706]; the 10.13039/501100001809National Natural Science Foundation of China [grant numbers 81730029, 81873704, 81900953, 81870731]; and the Beijing Natural Science Foundation [grant numbers 7191011, 7192234].

## CRediT authorship contribution statement

**Dejun Zhang:** Writing – original draft. **Jie Wu:** Investigation. **Yongyi Yuan:** Data curation. **Xiaohong Li:** Methodology. **Xue Gao:** Formal analysis. **Dongyang Kang:** Funding acquisition. **Xin Zhang:** Validation. **Sha-sha Huang:** Conceptualization. **Pu Dai:** Supervision.

## Declaration of competing interest

The authors declare that they have no known competing financial interests or personal relationships that could have appeared to influence the work reported in this paper.

## Abbreviations

transfer RNA: tRNA
ND: NADH-ubiquinone oxidoreductase chain
mitochondrial DNA: mtDNA
sensorineural hearing loss: SNHL;
next-generation sequencing: NGS
Burrows-Wheeler Aligner: BWA
Genome Analysis Toolbox: GATK
Variant Effect Predictor: VEP
Exome Aggregation Consortium: ExAC
Genome Aggregation Database: gnomAD
the Human Gene Mutation Database: HGMD
polymerase chain reaction: PCR
Aminoglycoside antibiotics: AmAn
H: human
B: bovine;
M: mouse
X: Xenopus laevis
rCRS: revised Cambridge Reference; Sequences
CO: cytochrome *c* oxidase
CytB: Cytochrome B
ATP6: ATP synthase membrane subunit 6
Thr: threonine;
Ala: alanine;
Ile: isoleucine;
Gly: glycine;
Pro: proline;
Leu: leucine;
Val: valine;
Asp;: aspartic acid
Met,: methionine;
Ser: serine;
PPK: palmoplantar keratoderma

## Footnotes

Appendix A Supplementary data to this article can be found online at https://doi.org/10.1016/j.heliyon.2024.e27041.
