# Supplementary material for: Mitochondrial tRNASer(UCN) mutations associated non-syndromic sensorineural hearing loss in Chinese families
Source: Heliyon. 2024 Mar 6;10(6):e27041. doi: 10.1016/j.heliyon.2024.e27041 (PMC10945119; doi:10.1016/j.heliyon.2024.e27041)
Supplement: Multimedia component 1 [file mmc1.doc]

Supplementary Table 1 168 deafness gene list

| ACTG1 | ALX3 | BSND | CABP2 | CCDC50 | CDH23 | CEACAM16 |
| --- | --- | --- | --- | --- | --- | --- |
| CHD7 | CIB2 | CLDN14 | CLPP | CLRN1 | COCH | COL11A1 |
| COL11A2 | COL1A1 | COL1A2 | COL2A1 | COL4A3 | COL4A4 | COL4A5 |
| COL4A6 | COL9A1 | COL9A2 | CRYM | DFNA5 | DFNB31 | DFNB59 |
| DIABLO | DIAPH1 | DIAPH3 | DSPP | ECM1 | EDN3 | EDNRB |
| ELMOD3 | ESPN | ESRRB | EYA1 | EYA4 | FGF3 | FGF8 |
| FGFR1 | FGFR3 | FLNA | FOXI1 | FREM1 | FXN | GATA3 |
| GIPC3 | GJB1 | GJB2 | GJB3 | GJB6 | GLYAT | GPR98 |
| GPSM2 | GRHL2 | GRXCR1 | HARS | HARS2 | HGF | HMX1 |
| HOXA2 | HSD17B4 | IL13 | ILDR1 | KARS | KCNE1 | KCNJ10 |
| KCNQ1 | KCNQ4 | KITLG | KRT9 | LAMA3 | LARS2 | LHFPL5 |
| LOXHD1 | LRTOMT | MARVELD2 | MIR96 | MITF | MPZ | MSRB3 |
| MYH14 | MYH9 | MYO15A | MYO1A | MYO1E | MYO3A | MYO6 |
| MYO7A | NDP | NDRG1 | NEFL | NELL2 | NF2 | OPA1 |
| OTOA | OTOF | OTOG | OTOGL | P2RX2 | PABPN1 | PAX3 |
| PCDH15 | PCDH9 | PDZD7 | PMP22 | PNPT1 | POLR1C | POLR1D |
| POU3F4 | POU4F3 | PROK2 | PROKR2 | PRPS1 | PTPN11 | PTPRQ |
| PTPRR | RDX | RPGR | SALL1 | SALL4 | SEC23A | SEMA3E |
| SERPINB6 | SIX1 | SIX5 | SLC17A8 | SLC19A2 | SLC26A4 | SLC26A5 |
| SMAD4 | SMPX | SNAI2 | SOX10 | STRC | TBC1D24 | TCIRG1 |
| TCOF1 | TECTA | TIMM8A | TJP2 | TMC1 | TMEM126A | TMIE |
| TMPRSS3 | TMPRSS4 | TNC | TPRN | TRIOBP | TRMU | TSPEAR |
| TYR | USH1C | USH1G | USH2A | WFS1 | MT-RNR1 | MT-TL1 |
| MT-CO1 | MT-TS1 | MT-TK | MT-TE | miR-96 | miR-182 | miR-183 |

Supplementary Table 2. Summary of the clinical data for matrilineal subjects in Family 1

| **No.** | **Subject**  **ID** | **Gender** | **Age**  **(years)** | **Age at onset**  **(years)** | **PTA (dB) right/left ear** | **Level of**  **hearing loss** | **PPK** | **Use of**  **AmAn.** |
| --- | --- | --- | --- | --- | --- | --- | --- | --- |
| 1 | III:1 | M | 60 | 17 | 105/106 | Profound | Yes | No |
| 2 | III:3 | M | 56 | 12 | 80/91 | Severe | No | No |
| 3 | III:5 | F | 56 | 15 | 80/85 | Severe | No | No |
| 4 | III:9 | F | 60 | 10 | 117/116 | Profound | No | No |
| 5 | III:10 | F | 57 | 9 | 112/113 | Profound | No | No |
| 6 | III:12 | F | 56 | 16 | >120/116 | Profound | No | No |
| 7 | III:14 | F | 53 | 13 | 76/88 | Severe | No | No |
| 8 | IV:4 | M | 34 | <1 | 105/100 | Profound | No | No |
| 9 | IV:6 | F | 31 | 8 | 112/110 | Profound | No | No |
| 10 | IV:8 | F | 33 | 20 | 67/57 | Moderately  severe | Yes | No |
| 11 | IV:17 | F | 30 | 9 | 103/95 | Profound | No | No |
| 12 | IV:19 | M | 29 | 10 | 85/95 | Severe | Yes | No |
| 13 | IV:20 | F | 26 | 6 | 85/87 | Severe | No | No |
| 14 | V:3 | F | 11 | 5 | 47/41 | Moderate | Yes | No |
| 15 | V:4 | M | 9 | 6 | 33/32 | Mild | No | No |
| 16 | V:5 | M | 9 | - | 18/18 | Normal | No | No |
| 17 | V:6 | F | 7 | - | 16/16 | Normal | No | No |
| 18 | V:9 | F | 10 | <1 | 83/66 | Moderately  severe | Yes | No |
| 19 | V:10 | M | 5 | <1 | 90/100 | Profound | No | No |
| 20 | V:12 | M | 9 | <1 | 85/91 | Severe | No | No |
| 21 | V:13 | M | 5 | <1 | 92/87 | Severe | No | No |

PTA, pure-tone audiometry; dB, decibel; PPK, palmoplantar keratoderma.

Supplementary Table 3 Summary of the clinical data for subjects in Family 2

| **No.** | **Subject**  **ID** | **Gender** | **Age(y)** | **Age at of onset(y)** | **PTA (dB) right /left ear** | **Level of hearing loss** | **Use of**  **AmAn.** |
| --- | --- | --- | --- | --- | --- | --- | --- |
| **1** | II:5 | F | 57 | 6 | 96/90 | Profound | No |
| **2** | II:11 | M | 50 | 4 | 86/112 | Profound | No |
| **3** | II:12 | F | 44 | After birth | >120/>120 | Profound | No |
| **4** | III:5 | F | 33 | 12 | 78/81 | Severe | No |
| **5** | III:11 | F | 24 | - | 20/15 | Normal | No |
| **6** | IV:1 | F | 24 | 6 | 96/95 | Profound | Yes |
